# Development and Application of a CAFLUX HepG2 Reporter Cell Line for Real-Time Monitoring of AhR-Mediated CYP1A1 Gene Expression in Response to Environmental Toxicants and Bioactive Modulators

**DOI:** 10.3390/ijms262010029

**Published:** 2025-10-15

**Authors:** Huyen Thi La, Hanh Hong Hoang, Phuc Minh Thi Le, Linh Thuy Nguyen, Da Thi Nguyen, Van Hanh Nguyen, Tam Minh Thi Ha, Long Hoang Nguyen, Dat Tien Nguyen

**Affiliations:** 1 Institute of Biology, Vietnam Academy of Science and Technology, Hanoi 100000, Vietnam; 2 Faculty of Biology, Graduate University of Science and Technology, Vietnam Academy of Science and Technology, Hanoi 100000, Vietnam; 3 Faculty of Agricultural Technology, VNU University of Engineering and Technology, 144 Xuan Thuy, Cau Giay, Hanoi 100000, Vietnam; 4 Faculty of Early Childhood Education, HaNoi Pedagogical University 2, 32 Nguyen Van Linh, Xuan Hoa 290000, Vietnam; 5 Institute for Advanced Study in Technology, Ton Duc Thang University, Ho Chi Minh City 70000, Vietnam; 6 Faculty of Pharmacy, Ton Duc Thang University, Ho Chi Minh City 70000, Vietnam; 7 Center for High Technology Research and Development, Vietnam Academy of Science and Technology, Hanoi 100000, Vietnam

**Keywords:** AhR signaling, CYP1A1, CAFLUX, HepG2, TCDD, benzo[a]pyrene, curcumin, extracellular vesicles, reporter gene assay, toxicogenomics

## Abstract

This study reports the construction and validation of a CAFLUX (Chemically Activated Fluorescent Expression) HepG2 reporter cell line engineered to express a histone H2B–green fluorescent protein (H2B–GFP) fusion protein under the control of a dioxin-responsive cytochrome P450 1A1 (CYP1A1) promoter. A lentiviral construct containing a synthetic promoter with multiple dioxin-responsive elements (DREs) upstream of the H2B–EGFP coding sequence was cloned into the pFUGW vector, packaged in human embryonic kidney (HEK) 293FT cells, and used to transduce HepG2 hepatocellular carcinoma cells. Stable clones obtained by limiting dilution were screened for GFP expression in response to 2,3,7,8-tetrachlorodibenzo-p-dioxin (TCDD). The resulting CAFLUX HepG2 cells exhibited dose-dependent nuclear GFP fluorescence when exposed to aryl hydrocarbon receptor (AhR) agonists, with limits of detection of approximately 0.01 pM for TCDD and 0.1 pM for benzo[a]pyrene (B[a]P), a polycyclic aromatic hydrocarbon (PAH). This reporter activity correlated with endogenous CYP1A1 mRNA expression as determined by quantitative polymerase chain reaction (qPCR), confirming that GFP signals reflected native transcriptional responses. In functional assays, curcumin suppressed GFP expression in a concentration-dependent manner and induced apoptotic morphology at higher doses, while extracellular vesicles (EVs) derived from adipose-derived stem cells (ADSCs) significantly reduced both GFP fluorescence and CYP1A1 mRNA levels, suggesting an inhibitory effect on AhR-driven transcription. The CAFLUX HepG2 reporter system therefore provides a sensitive and reproducible platform for real-time, nuclear-localized monitoring of AhR-mediated gene expression. Its responsiveness to both agonists and antagonists underscores its potential utility in toxicological evaluation, drug discovery, and the investigation of EV-mediated signaling in liver cancer models.

## 1. Introduction

Dioxins, particularly 2,3,7,8-tetrachlorodibenzo-p-dioxin (TCDD), are among the most toxic environmental pollutants and are classified as persistent organic pollutants (POPs). These compounds are widely distributed in the environment and bioaccumulate through the food chain, particularly in the fatty tissues of animals. According to the World Health Organization (WHO), over 90% of human exposure to dioxins occurs through dietary intake—primarily from meat, dairy products, fish, and shellfish. Dioxins are associated with a wide range of adverse health effects, including reproductive and developmental disorders, immune suppression, endocrine disruption, and cancer [1].

The toxicological effects of dioxins are primarily mediated by the aryl hydrocarbon receptor (AhR), a ligand-activated transcription factor. Upon binding to ligands such as TCDD, AhR translocates to the nucleus, dimerizes with the AhR nuclear translocator (ARNT), and binds to dioxin-responsive elements (DREs) in the promoter regions of target genes. Activation of AhR leads to induction of xenobiotic-metabolizing enzymes, particularly CYP1A1 and CYP1B1, which are central to the biotransformation of foreign compounds and maintenance of cellular homeostasis. However, sustained or dysregulated induction of these enzymes can generate reactive metabolites and oxidative stress, thereby contributing to tissue injury and carcinogenesis [2,3]. However, chronic AhR activation by dioxins has been linked to sustained immunosuppression and increased risk of tumorigenesis, particularly in the liver [4,5,6].

As the central organ for xenobiotic metabolism, the liver is particularly vulnerable to dioxin-induced toxicity. Several studies have demonstrated that TCDD causes hepatocellular injury and markedly alters gene expression in both liver tissues and cell-based models. Upregulation of enzymes such as CYP1A1, CYP1B1, and anterior gradient 2 (AGR2) contributes to inflammatory responses and hepatocarcinogenesis [7,8,9,10,11]. The HepG2 human liver cancer cell line has therefore become a widely accepted in vitro model for evaluating hepatic toxicity and exploring the mechanisms underlying dioxin-induced apoptosis, necrosis, and metabolic alterations [7,8,12,13,14,15].

In addition to dioxins, other polycyclic aromatic hydrocarbons (PAHs) can also activate the aryl hydrocarbon receptor (AhR) and exert comparable toxic effects. Benzo[a]pyrene (B[a]P) is a typical example. This compound is generated during incomplete combustion processes and is present in cigarette smoke, vehicle exhaust, industrial emissions, and charred foods. B[a]P is considered one of the most toxic PAHs; it is metabolized by the cytochrome P450 system into reactive epoxides that form DNA adducts and induce oxidative stress, thereby contributing to carcinogenesis [16]. Recent studies further indicate that B[a]P exposure triggers strong inflammatory responses in the lungs, alters the composition of alveolar epithelial and macrophage cells, enhances reactive oxygen species production, and upregulates antioxidant genes [16]. Beyond its carcinogenic potential, B[a]P has also been linked to cardiovascular disease, immunosuppression, reproductive and respiratory disorders, gastrointestinal dysfunction, and DNA damage [17]. Therefore, research and monitoring of B[a]P alongside dioxins are essential for elucidating AhR-mediated toxic mechanisms and developing appropriate control strategies.

A major advancement in dioxin detection has been the development of cell-based reporter assays, particularly the CALUX (Chemically Activated LUciferase gene eXpression) system. This technique employs engineered cells that express a luciferase gene under control of the CYP1A1 promoter, enabling sensitive detection of AhR pathway activation via luminescence. CALUX assays can detect TCDD concentrations as low as 0.1 pM and are used to estimate the total toxic equivalent (TEQ) of dioxin-like compounds in environmental samples [18,19,20,21].

Over the past decade, efforts to optimize CALUX have focused on three main strategies: (1) increasing the number of dioxin-responsive elements (DREs) in reporter vectors; (2) improving the luciferase gene constructs; and (3) utilizing next-generation luciferases with enhanced sensitivity. Several commercial CALUX cell lines now achieve detection limits as low as 0.1–1 pM, with stable responses lasting up to 48 h. However, most commercial CALUX lines rely on transient transfection, making them prone to plasmid loss and signal degradation over successive passages. This instability reduces reproducibility and increases costs due to the need for frequent replacement. Moreover, luciferase-based assays require cell lysis and substrate addition, which may introduce technical variability. Importantly, luciferase does not allow real-time monitoring of reporter activity in living cells, limiting its utility for continuous or dynamic analyses [22,23,24,25].

In addition to environmental toxins, certain bioactive compounds have shown potential to modulate AhR signaling. One such compound is curcumin, a polyphenol extracted from *Curcuma longa*, known for its anti-inflammatory, antioxidant, and anti-cancer properties. Curcumin has been reported to influence the AhR-CYP1A1 axis, potentially suppressing xenobiotic-induced gene expression [26]. Similarly, extracellular vesicles (EVs) derived from adipose-derived stem cells (ADSCs) are nanostructures capable of modulating gene expression and intercellular signaling. Although EVs exhibit anti-tumor and anti-inflammatory effects, their role in regulating AhR activity and CYP1A1 expression remains poorly characterized [11].

Despite the effectiveness of luciferase-based CALUX systems in detecting dioxins and evaluating AhR activation, there remains a significant gap in the availability of fluorescence-based reporter models that allow real-time, spatial visualization of AhR signaling and its downstream gene expression, particularly in human hepatocellular carcinoma cells. Moreover, while emerging studies suggest that curcumin and ADSC-derived extracellular vesicles (EVs) may influence xenobiotic metabolism and inflammatory pathways, their direct impact on AhR-mediated transcriptional responses—especially CYP1A1 gene regulation—has not been systematically investigated. A robust, cell-based fluorescent reporter platform would thus provide a valuable tool to assess both environmental toxicants and therapeutic modulators with precision and reproducibility.

To address these gaps, this study aimed to construct and validate a novel CAFLUX HepG2 reporter cell line expressing an H2B-GFP fusion protein under the control of a DRE-containing CYP1A1 promoter, enabling real-time visualization of AhR pathway activation. This system was then applied to evaluate the effects of TCDD, benzo[a]pyrene (B[a]P), curcumin, and ADSC-derived EVs on CYP1A1 expression and AhR signaling activity.

To address these gaps, the study aimed to construct and validate a stable CAFLUX reporter cell line derived from HepG2, in which a fusion between the gene encoding histone H2B and the EGFP gene (H2B-EGFP) is placed under the control of a CYP1A1 promoter containing dioxin-responsive elements (DREs). When DRE ligands are present, the H2B-EGFP fusion proteins are expressed and accumulate in the cell nucleus, allowing real-time monitoring of AhR activation. This system was then used to assess the effects of TCDD, benzo[a]pyrene (B[a]P), curcumin and extracellular vesicles (EVs) from adipose-derived stem cells on CYP1A1 expression and AhR signaling activity.

## 2. Results

### 2.1. Results of Vector Construction and Generation of CAFLUX HepG2 Cell Line: Limiting Dilution and Fluorescence Microscopy

**Figure 1:**
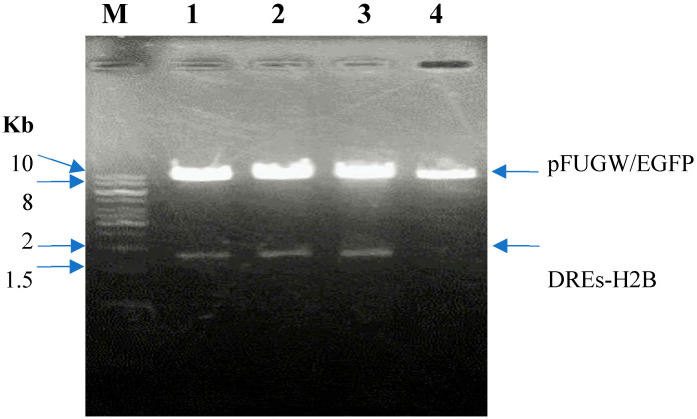
Agarose gel electrophoresis of pFUGW/DREs-H2B-EGFP gene construct Digested with PacI and BamHI.

The pFUGW vector (~9955 bp, Addgene #14883), a lentiviral backbone containing the EGFP gene, was selected as the cloning scaffold. The DRE-containing promoter and H2B gene were excised from the donor plasmid DREs-H2B (Addgene #182294) using the restriction enzymes *Pac*I and *Bam*HI and inserted into the linearized pFUGW vector. The recombinant plasmid was then digested and verified by 1% agarose gel electrophoresis. The gel electrophoresis result is shown in Figure 1.

Lane M: 1 kb DNA ladder (Thermo Fisher Scientific, Waltham, MA, USA); Lane 1-3: Digested pFUGW/DREs-H2B-EGFP construct clone 1-3 with *Pac*I and *Bam*HI; 4: Digested pFUGW/EGFP with *Pac*I and *Bam*HI.

The digestion of recombinant pFUGW containing the DRE promoter and H2B-EGFP coding sequence yielded two bands of approximately 1500 bp and 10 kb, which closely correspond to the theoretical sizes of 1646 bp and 9950 bp, confirming successful ligation. The recombinant plasmids were then sequenced to verify the insert as below:
1 ATTATTTTCTGGCCTGGACCAGCGACGGATGGAGGTGCCACCGGGTTGGGGAGCACGTCG61 GGGATGGCGCGTAACGATGTTAGCTGGGGCCAGGTTGAGCTAGGCACGCAAATACAACTT121 TTTTTTTCCTGGAAACCCTGTAACAGGAAGGTTCCGGAGGGCGGGACAGCGTCGGAGGCA181 GGCAGCTAGGCCATGCCAAATGGCACTGGGGCTTCGTGTCGTGCCACAGGCGTGGACCGA241 AAATGCGGACACATGCAGGCTGCCTCTCCTCGCAGGCAGAAGCCACACGCAGACCTAGAC301 CCTTTGCACCGCATCCCCTTATTCAATCGCGCACCCGCCACCCTTCGACAGTTCCTCTCC361 CTCCACCCCAACCCCACGCCGCGCGCGAGGCTGGCCCTTTAAGAGCCCCGCCCCGACTCC421 CTCCCCCCTCGCGTGACTGCGAGCCCCCGCGCCGGGCCGGGGAATGGGTCGGCTGGGTGG481 CTGCGCGGGCCTCCGGTCCTTCTCACGCAACGCCTGGGCACCGCGCCTCCGGGCCAGGTG541 GGGCGGGGACGGGCCGCCTGACCTCTGCCCCCTAGAGGGATGTCGCCGGCGCACGCAAGC601 TAGCCGGGGGTAGGGTGGGGGCTCCGCGCCAGGTGCCCCCTCCGTGGTCCCTGGGCCCGA661 GTCTTTCCGTGGCCCCCCGCCGCCGGATTTCTGTGCTCTGCCAATCAAAGCACTAGCCAC721 CCCGGGAGCCAAGAGGGACCCTCAAGGGCCGGTGGGTCCTGGCTGGAGGGACCGCGCGTT781 GCAATCAGCACTAAGGCGATCCTAGAGGCTGCGAGGAGCCGCTAGTGAGCGCTCAGCGAG841 CCTGCCCCTTCGCCATCCATTCCGATCCTTCAATCAAGAGGCGCGAACCTCAGCTAGTCG901 CCCGGGCTCTGGGGGACAGGTCCAGCCCCGCGGCGCCTCTGGCCTTCCGGCCCCCGTGAC961 CTCAGGGCTGGGGTCGCAGCGCTTCTCACGCGAGCCGGGACTCAGTAACCCCGGGAAGAA1021 GGTCACCACGGGGCAGCCCCGCCCCCGCCTGCCGAGTCCTGGTAGGCTGTAGCGCTGGGG1081 AGGCATCTGCACGCCCAGCGTTCCAGTGGGTGCAAAAATGACGAAGAGGAGTCCCCGCGC1141 CCCAGGATGGAGCTTCCCGTACCCTCTCTTCGGGCTGTCCTGGGACTTCTCCCTCAAGCC1201 CCCTCCTCGGCTGGGTTCTGCACTGCCCTTGGGACGCCTTGGAATTGGGACTTCCAGGTG1261 TTCCCAGCCCTCACCCCTCTATGTACAGGCACCGAGATGTGTCCCATAGTGGGTTCTTGC1321 CCACCCGACCCCCCACCCCCGCCGCCCTCCGCCACCTTTCTCTCCAATCCCAGAGAGACC1381 AGCCCGGTTCAGGCTGCTTCTCCCTCCATCTCAGCTCGCTCCAGGGAAGGAGGCGTGGCC1441 ACACGTACAAGCCCGCCTATAAAGGTGGCAGTGCCTTCACCCTCACCCTGAAGGTGACAG1501 TTCCTTGGAACCTTCCCTGATCCTTGTGATCCCAGGCTCCAAGAGTCCACCCTTCCCAGC1561 TGAGCTCAGATCTCGAGCTCAAGCTTCGAATTCTGCAGTCGACGGTACCGCCACCATGGT1621 GAGCAAGGGCGAGGAGCTGTTCACCGGGGTGGTGCCCATCCTGGTCGAGCTGGACGGCGA1681 CGTAAACGGCCACAAGTTCAGCGTGTCCGGCGAGGGCGAGGGCGATGCCACCTACGGCAA1741 GCTGACCCTGAAGTTCATCTGCACCACCGGCAAGCTGCCCGTGCCCTGGCCCACCCTCGT1801 GACCACCCTGACCTACGGCGTGCAGTGCTTCAGCCGCTACCCCGACCACATGAAGCAGCA1861 CGACTTCTTCAAGTCCGCCATGCCCGAAGGCTACGTCCAGGAGCGCACCATCTTCTTCAA1921 GGACGACGGCAACTACAAGACCCGCGCCGAGGTGAAGTTCGAGGGCGACACCCTGGTGAA1981 CCGCATCGAGCTGAAGGGCATCGACTTCAAGGAGGACGGCAACATCCTGGGGCACAAGCT2041 GGAGTACAACTACAACAGCCACAACGTCTATATCATGGCCGACAAGCAGAAGAACGGCAT2101 CAAGGTGAACTTCAAGATCCGCCACAACATCGAGGACGGCAGCGTGCAGCTCGCCGACCA2161 CTACCAGCAGAACACCCCCATCGGCGACGGCCCCGTGCTGCTGCCCGACAACCACTACCT2221 GAGCACCCAGTCCGCCCTGAGCAAAGACCCCAACGAGAAGCGCGATCACATGGTCCTGCT2281 GGAGTTCGTGACCGCCGCCGGGATCACTCTCGGCATGGACGAGCTGTACAAGTAA

The obtained sequence confirmed the correct integration of the DRE promoter, H2B gene, and EGFP coding region, preserving key regulatory and expression elements such as the open reading frame, Kozak sequence, and start codon, ensuring efficient transcription and translation of the H2B-EGFP fusion protein.

The validated plasmid pFUGW/DREs-H2B-EGFP, along with packaging plasmids pCMV-dR8.2dvpr (8455 bp) and pCMV-VSV-G (8454 bp), were purified using the QIAgen Plasmid Midiprep Kit (Germantown, MD, USA) and used to produce lentiviral particles in HEK 293FT cells (Thermo Fisher Scientific, Waltham, MA, USA).

### 2.2. Generation and Selection of CAFLUX HepG2 Cells

**Figure 2:**
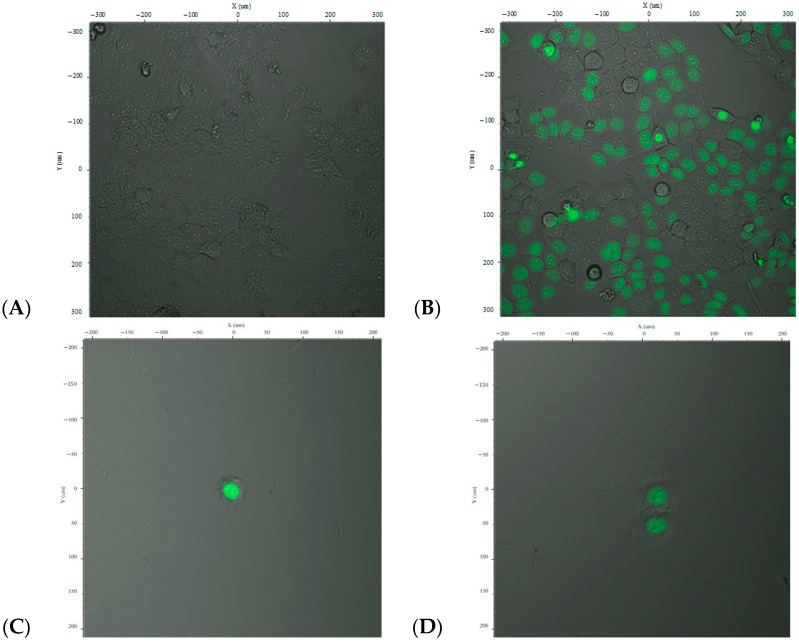

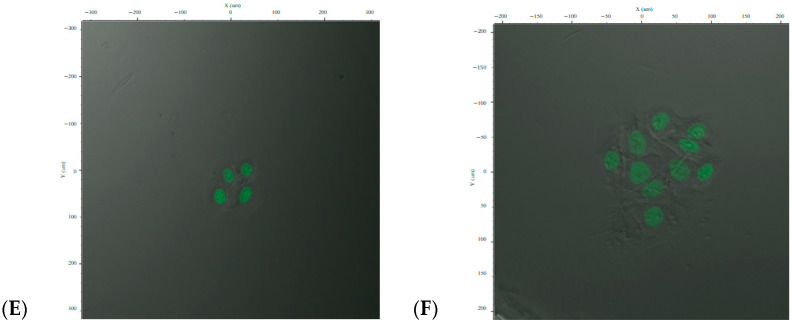
Fluorescence microscopy images of HepG2 cells transduced with the DREs-H2B-EGFP construct, showing control cells (**A**), induced transduced cells (**B**), and selected single-cell clones (**C**–**F**). The green fluorescence shows that the EGFP–histone fusion protein is expressed and localized in the nucleus.

The resulting lentiviral particles carrying pFUGW/DREs-H2B-EGFP were used to transduce HepG2 cells using OptiMEM medium (Carlsbad, CA, USA). Post-transduction, the cells were stimulated with TCDD (2,3,7,8-tetrachlorodibenzo-p-dioxin) to assess inducible expression. Fluorescence microscopy images of the transduced cells at varying magnifications are presented in Figure 2A,B.

Cells were examined under a fluorescence microscope using an excitation wavelength of 485 nm and emission wavelength of 515 nm. The images revealed both fluorescent (green-emitting) and non-fluorescent cells. The green fluorescence was observed in the nuclei, indicating expression of the H2B-EGFP fusion protein, confirming successful gene transfer and expression driven by the DRE promoter upon dioxin stimulation.

Analysis of the GFP fluorescence indicated a transfection efficiency of approximately 70–80%, depending on the cell population and field of view. However, as shown in Figure 2, the expression was not uniform across all cells, and full transduction efficiency was not achieved.

To isolate a homogenous population, cells were subjected to limiting dilution to establish individual CAFLUX HepG2 clones that stably express the H2B-EGFP fusion protein in response to dioxin exposure (Figure 2C–F).

### 2.3. Response of CAFLUX HepG2/DREs-H2B-EGFP Cells to Standard Compounds 2,3,7,8-TCDD and B[a]P

**Figure 3:**
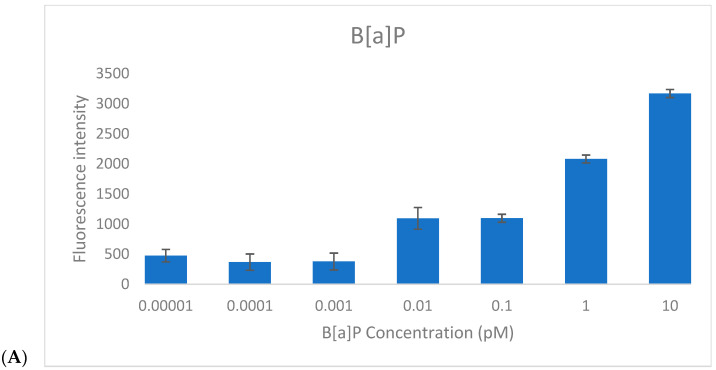

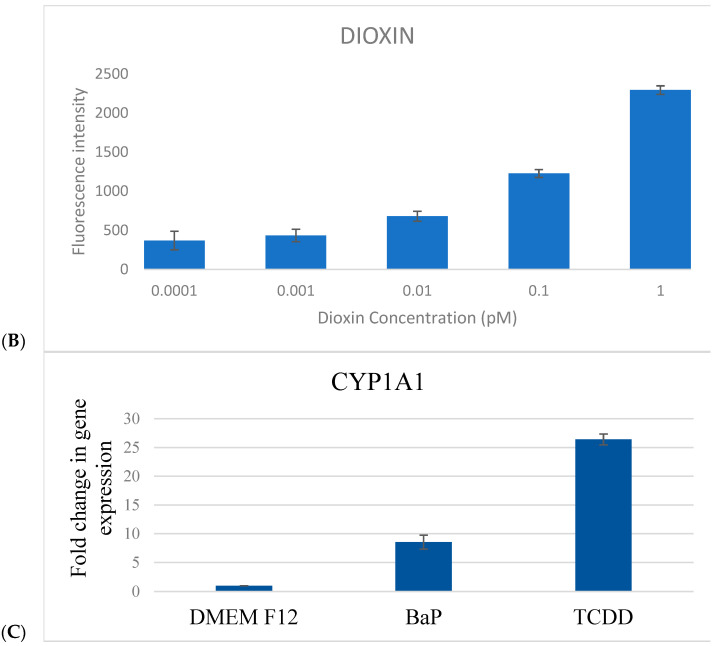
Fluorescence response of CAFLUX HepG2/DREs-H2B-EGFP cells after 24 h exposure to standard compounds. (**A**) Fluorescence signal after treatment with B[a]P at various concentrations; (**B**) Fluorescence signal after treatment with TCDD at various concentrations. Concentration values are displayed in logarithmic scale (LogX); (**C**) Quantitative PCR analysis of CYP1A1 mRNA expression in cells treated with B[a]P and TCDD at corresponding concentrations.

To assess the responsiveness of the CAFLUX HepG2/DREs-H2B-EGFP reporter cell line, cells were treated for 24 h with varying concentrations of benzo[a]pyrene (B[a]P, 0.00001–10 pM) and 2,3,7,8-tetrachlorodibenzo-p-dioxin (TCDD, 0.0001–1 pM), and GFP fluorescence intensity was quantified (Figure 3).

For B[a]P, a clear linear increase in fluorescence intensity was observed within the concentration range of 0.1–10 pM, indicating effective dose-dependent activation of the AhR pathway. The estimated limit of detection (LOD) for B[a]P was approximately 0.1 pM, based on the lowest concentration at which consistent fluorescence induction was recorded.

For TCDD, a similar concentration-dependent trend was noted. At very low levels (0.0001–0.001 pM), only minimal fluorescence variation was detected, but a progressive increase in GFP signal was evident from 0.001 pM to 1 pM. This suggests that higher TCDD concentrations may be required to fully determine the saturation point of the reporter system. The detection threshold for TCDD was established at ~0.01 pM, confirming the high sensitivity of the CAFLUX assay.

Importantly, fluorescence responses to TCDD were markedly stronger than those elicited by B[a]P at equivalent concentrations. This enhanced response likely reflects the higher binding affinity and stronger toxicological potency of TCDD toward the aryl hydrocarbon receptor (AhR), resulting in more robust activation of the DRE-driven H2B-EGFP expression.

Together, these findings validate the functionality and sensitivity of the CAFLUX HepG2/DREs-H2B-EGFP cell line as a reliable in vitro platform for detecting and comparing AhR ligands, including dioxins and polycyclic aromatic hydrocarbons (PAHs).

### 2.4. CYP1A1 mRNA Expression

To validate the biological relevance of the CAFLUX system at the transcriptional level, CYP1A1 gene expression was quantified using real-time RT-qPCR, with GAPDH serving as the internal reference. CAFLUX HepG2 cells were exposed to TCDD (1 pM) or B[a]P (1 pM) for 24 h before RNA extraction.

TCDD treatment resulted in a robust upregulation of CYP1A1 mRNA, showing a 26.4-fold increase relative to the untreated control (*p* < 0.001). B[a]P also significantly elevated CYP1A1 expression, though to a lesser extent, with an 8.6-fold increase (*p* < 0.01) (Figure 3C). These results align with the greater potency of TCDD in activating the aryl hydrocarbon receptor (AhR) pathway, as observed in the CAFLUX fluorescence data.

Together, these findings demonstrate that the CAFLUX HepG2 reporter system not only provides a sensitive readout of AhR activation via fluorescence but also reliably mirrors transcriptional changes in downstream target genes, reinforcing its utility for functional toxicology and bioactivity screening of AhR ligands.

### 2.5. Analysis of the Effects of ADSC-Derived Extracellular Vesicles on CYP1A1 Gene Activity Using the CAFLUX HepG2/DREs-H2B-EGFP Cell Line

Extracellular vesicles (EVs) were isolated from adipose-derived stem cell (ADSC) cultures as described in the Materials and Methods Section, followed by particle size distribution analysis and functional assays.

**Figure 4:**
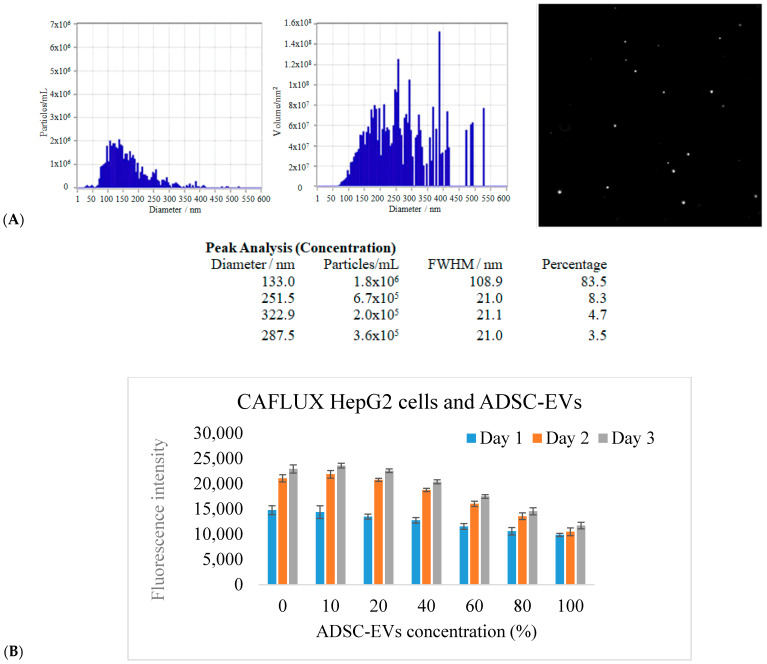
(**A**) ADSC-derived EVs and (**B**) GFP fluorescence intensity of CAFLUX HepG2/DREs-H2B-EGFP cells in response to ADSC-derived EVs.

Nanoparticle Tracking Analysis (NTA) revealed that the EVs exhibited a predominant size distribution between 80 and 200 nm, with a major peak centered at 133.0 nm, representing 83.5% of total particles. The particle concentration was determined to be 1.8 × 10^6^ particles/mL. Minor peaks at 251.5 nm (8.3%), 287.5 nm (3.5%), and 322.9 nm (4.7%) were also observed, possibly indicating microvesicles or limited particle aggregation (Figure 4A).

Further characterization using the ZetaView^®^ PMX-230-S-488/660 system (Badalona, Spain) confirmed that the ADSC-derived EVs were predominantly spherical and consistent with the expected size range of small EVs such as exosomes. Fluorescence mode imaging demonstrated a homogeneous particle distribution and validated the presence of fluorescently labeled vesicles. These findings confirm the successful isolation of intact EVs suitable for downstream bioactivity studies.

To evaluate the regulatory effects of ADSC-derived EVs on aryl hydrocarbon receptor (AhR)-mediated CYP1A1 gene activity, the CAFLUX HepG2/DREs-H2B-EGFP reporter cell line was utilized. Cells were seeded at equal densities in 96-well plates and treated with a gradient of EV concentrations (0% to 100% *v*/*v* in serum-free medium). After 24 h of incubation, nuclear-localized GFP fluorescence was quantified using a Biotek Synergy HT microplate reader (Winooski, VT, USA). The results demonstrated a dose-dependent reduction in GFP intensity, suggesting a suppressive effect of ADSC-EVs on AhR-driven CYP1A1 expression (Figure 4B).

Across all tested EV concentrations, a dose-dependent decrease in GFP fluorescence was observed, suggesting a suppression of AhR pathway activation. Despite the proliferative effect that ADSC-derived EVs are known to have on both HEK and HepG2 cells, their presence appeared to markedly reduce GFP expression, indicating a downregulation of CYP1A1 promoter activity. This initial finding suggested that the ADSC-derived EVs may attenuate xenobiotic-induced stress and reduce the expression of AhR-responsive genes.

To confirm these findings, co-culture experiments were conducted in which HepG2 cells were treated with ADSC-derived EVs and analyzed for CYP1A1 mRNA expression using real-time PCR.

**Figure 5:**
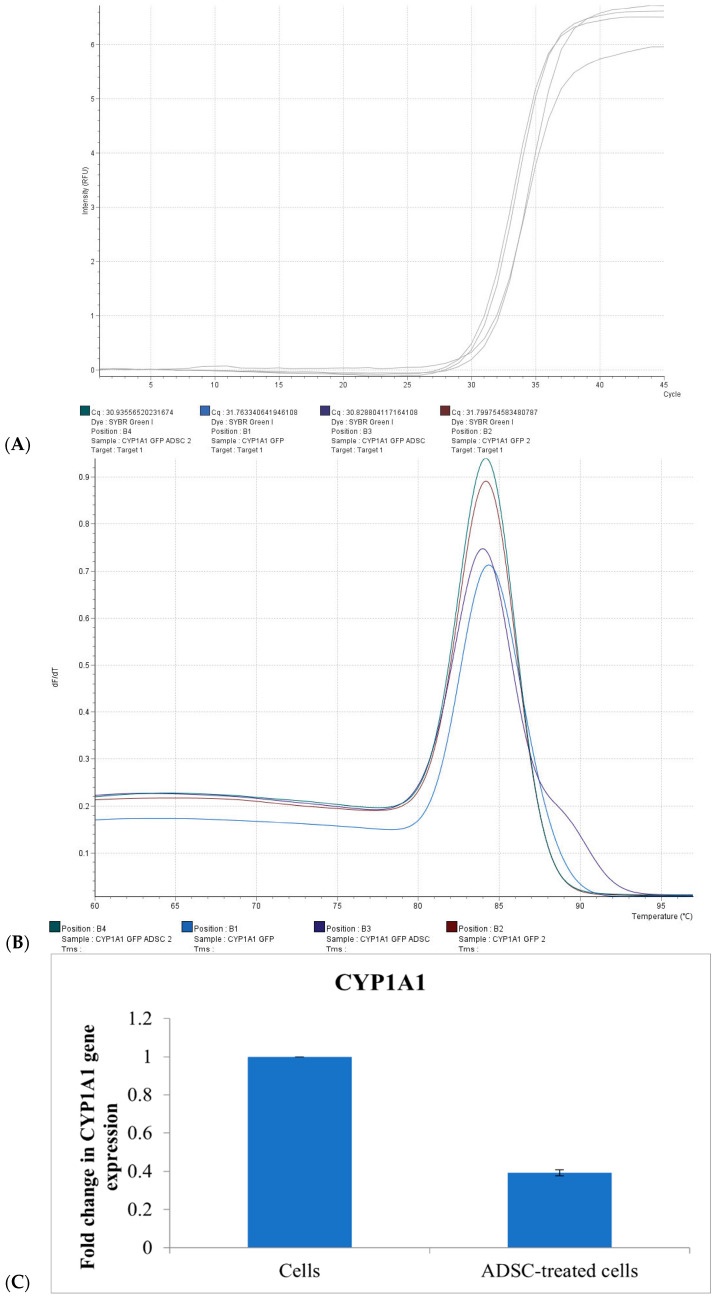
Real-time PCR analysis of CYP1A1 gene expression in CAFLUX HepG2 cells treated with ADSC-derived EVs. (**A**) Amplification plot of CYP1A1; (**B**) Melt curve analysis; (**C**) Fold change of CYP1A1 gene expression.

The amplification plot demonstrated a delayed fluorescence signal in EV-treated cells compared to untreated controls, indicating a lower level of CYP1A1 transcript. Melt curve analysis revealed a single specific peak at approximately 84 °C, confirming the specificity of the amplification. Untreated CAFLUX HepG2 control cells exhibited a lower Ct value, with a ΔΔCt for CYP1A1 of 1.409 ± 0.061 compared to EV-treated samples. No significant change in Ct value was observed in the group treated with ADSC-derived EVs, indicating suppressed gene expression (Figure 5).

Quantitative analysis revealed that culturing HepG2 cells in medium supplemented with 40% ADSC-derived EVs resulted in a 62.3% ± 1.6% reduction in CYP1A1 expression relative to the control group. These results support the use of the CAFLUX HepG2/DREs-H2B-EGFP reporter system as an effective tool for investigating the influence of external factors—including bioactive EVs—on CYP1A1 expression and AhR signaling pathway modulation.

### 2.6. Using the CAFLUX HepG2/DREs-H2B-EGFP Cell Line to Analyze the Effects of Extracellular Vesicles Derived from Adipose-Derived Stem Cells on CYP1A1 Gene Activity in Liver Cancer Cells

**Figure 6:**
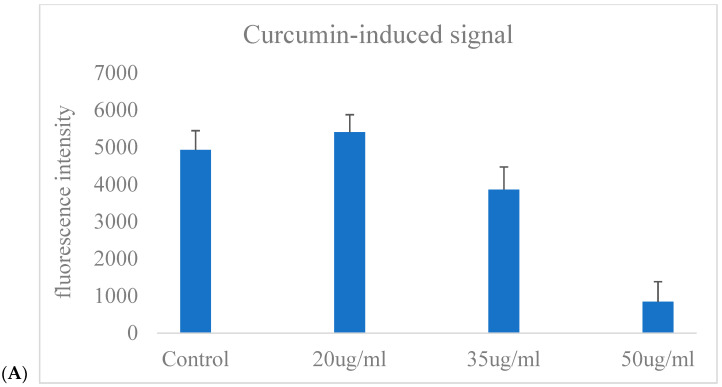

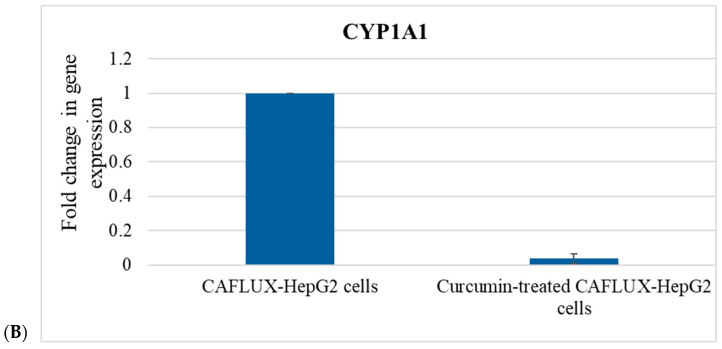
Effect of curcumin on the fluorescence signal of CAFLUX HepG2 cells (**A**) and analysis of Cyp1a1 gene expression (**B**).

Figure 6A illustrates the GFP fluorescence intensity of CAFLUX HepG2/DREs-H2B-EGFP cells following exposure to varying concentrations of curcumin (Cur). The fluorescence signal remained relatively unchanged in the control and slightly increased at 20 µg/mL Cur. However, a concentration-dependent decrease in fluorescence was observed at higher doses, with a marked reduction at 35 µg/mL and a dramatic suppression at 50 µg/mL. This result indicates that higher concentrations of curcumin may inhibit the AhR signaling pathway, leading to downregulation of the GFP reporter gene controlled by the DRE promoter. Figure 6A reflects the GFP fluorescence response to Cur exposure, demonstrating that curcumin reduces GFP expression, which correlates with decreased CYP1A1 gene activity in HepG2 cells.

To assess the impact of curcumin on aryl hydrocarbon receptor (AhR)-mediated transcriptional activity, CYP1A1 mRNA levels were measured by real-time PCR in CAFLUX-HepG2 cells following treatment with 30 μg/mL curcumin for 24 h. As illustrated in Figure 6B, curcumin treatment resulted in a substantial decrease in CYP1A1 transcript levels compared to untreated controls.

The fold change in gene expression was reduced to below 0.1, indicating strong suppression of CYP1A1 transcription. These findings suggest that curcumin functions as an AhR antagonist or transcriptional repressor, thereby inhibiting downstream gene activation. This transcriptional suppression is consistent with the concurrent decrease in GFP fluorescence observed in the CAFLUX system, further supporting curcumin’s potential role in modulating xenobiotic-responsive signaling pathways.

**Figure 7:**
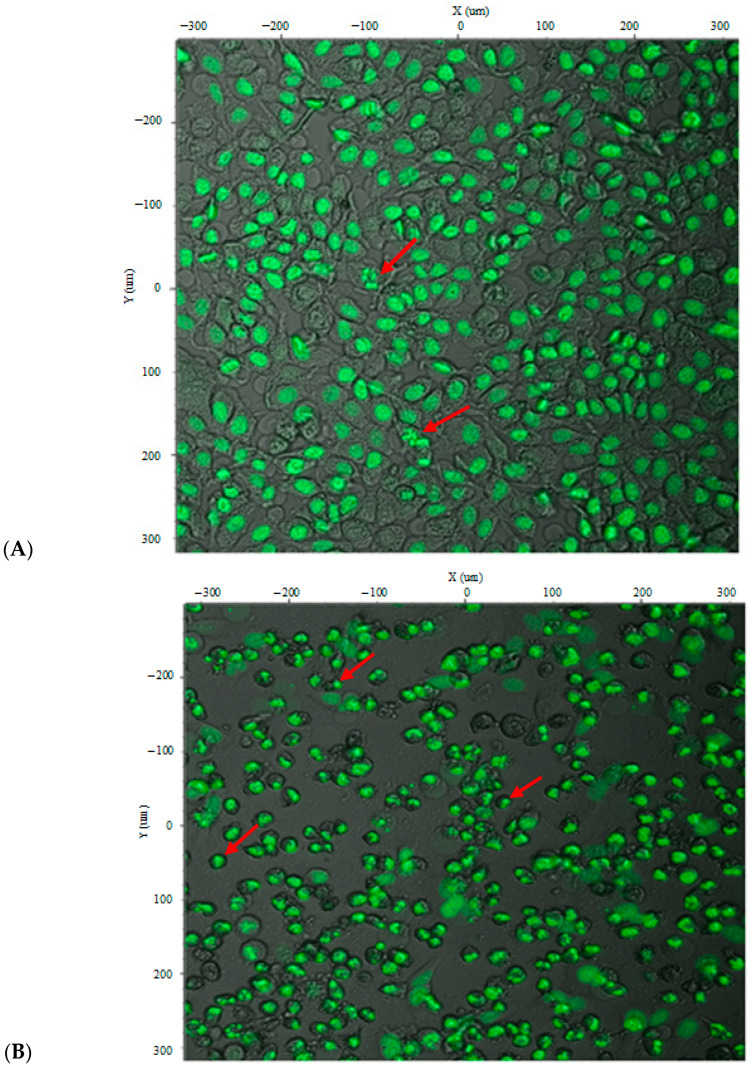
CAFLUX HepG2 cells treated with curcumin and imaged using a Nikon C1si confocal microscope. (**A**) At 35 µg/mL curcumin, the nuclei begin to fragment (red arrow). (**B**) At 50 µg/mL curcumin, chromatin fragments are packaged into apoptotic bodies (red arrow). The H2B–GFP fusion protein binds chromosomal DNA, remaining attached to DNA fragments during fragmentation. This allows clear visualization of DNA degradation and the formation of apoptotic bodies.

Figure 7 illustrates the morphological and nuclear changes in CAFLUX HepG2 cells expressing the H2B-GFP fusion protein after curcumin treatment at 35 µg/mL and 50 µg/mL. Because the H2B-GFP fusion localizes to chromatin, the nuclear architecture can be visualized directly. Curcumin treatment produced heterogeneous cell morphologies; many cells contracted and became rounded, suggesting early or ongoing apoptosis. Some nuclei appeared brighter and irregularly dispersed, consistent with nuclear fragmentation—a hallmark of apoptosis. These effects were more pronounced at the higher concentration (50 µg/mL), at which most cells were dead and chromatin fragments had been packaged into apoptotic bodies, highlighting curcumin’s cytotoxicity toward HepG2 cells. Together, these observations indicate that the CAFLUX HepG2 cell line can be used to analyze, at least in part, the mechanisms by which various agents induce death in HepG2 liver cancer cells.

## 3. Discussion

This study successfully established and validated a novel CAFLUX HepG2 reporter cell line engineered to express a nuclear-localized H2B-GFP fusion protein under the control of a dioxin-responsive CYP1A1 promoter. The CAFLUX system enabled real-time visualization of AhR-mediated transcriptional activity in human hepatocellular carcinoma cells. The reporter cells demonstrated dose-dependent responses to classical AhR agonists (TCDD and B[a]P) and were further used to evaluate the modulatory effects of bioactive agents such as curcumin and extracellular vesicles (EVs) derived from adipose-derived stem cells (ADSCs). These results confirmed the system’s utility for mechanistic toxicology and therapeutic screening.

The establishment of the CAFLUX HepG2 cell line represents a significant advancement in cell-based biosensor development for toxicological research. By fusing EGFP to histone H2B and linking its expression to a DRE-containing CYP1A1 promoter, we enabled both quantitative and spatial tracking of AhR activation. This approach complements traditional luciferase-based assays such as CALUX but adds the benefit of nuclear-localized fluorescence for high-resolution imaging. The dose-dependent fluorescence in response to TCDD and B[a]P is consistent with known mechanisms of AhR activation, where ligand binding triggers AhR-ARNT dimerization and binding to XREs, leading to CYP1A1 transcription [27,28,29]. Our data confirm that the CAFLUX system effectively captures these transcriptional dynamics in living HepG2 cells.

A key highlight of this study is the exceptionally high sensitivity of the CAFLUX system in detecting AhR activation signals, particularly for TCDD at concentrations as low as 0.01 pM—surpassing the commonly reported detection limits of traditional CALUX (luciferase-based) assays. Previous studies have shown that CALUX systems typically detect TCDD at limits ranging from 0.1 to 1 pM [22,30]. Therefore, the data obtained from CAFLUX suggest that it is a more sensitive and reliable tool for assessing the activity of AhR agonists.

The superior sensitivity of the system is primarily attributed to its optimized reporter design. The fusion of GFP with histone H2B ensures nuclear localization of the fluorescent signal, thereby enhancing contrast and resolution during image analysis. Moreover, unlike luciferase-based assays which require the addition of external substrates like luciferin, the CAFLUX system enables continuous, non-invasive real-time monitoring without any substrate supplementation. This provides a distinct advantage in detecting weak signals and studying the dynamics of live cells over time.

In addition, the stability and uniformity of the fluorescent signal from the CAFLUX cell line are critical factors that enhance the assay’s reliability. Following lentiviral transduction with a DRE-driven H2B–EGFP construct, monoclonal cell lines were selected using a limiting dilution method to eliminate heterogeneity in reporter expression. As a result, the recorded signals across experiments exhibited high reproducibility and accuracy, meeting the rigorous demands of mechanistic toxicology research and in vitro drug screening.

The response of the CAFLUX cells to curcumin adds an important layer to understanding how natural compounds can modulate AhR signaling. Curcumin significantly suppressed both GFP fluorescence and CYP1A1 gene expression in a dose-dependent manner, supporting its reported role as an AhR antagonist [26,28,31]. Mechanistically, curcumin is known to inhibit AhR nuclear translocation and XRE binding, thereby blocking the transcription of downstream targets like CYP1A1 [32]. These effects likely contribute to its observed anti-inflammatory and anti-tumor properties. By using CAFLUX, we provided direct visual confirmation of curcumin’s ability to suppress AhR activity at the nuclear level—highlighting the biosensor’s utility in evaluating both toxicants and therapeutic modulators.

In addition to transcriptional suppression, curcumin induced clear apoptotic features at higher concentrations, including nuclear condensation and fragmentation visualized through the H2B-GFP signal. These morphological changes are consistent with programmed cell death and suggest that curcumin’s cytotoxic effects in liver cancer may result from both transcriptional and post-transcriptional mechanisms [32]. The CAFLUX system allowed us to capture these apoptotic signatures alongside gene expression changes, offering an integrated platform for studying toxicity and therapeutic efficacy.

A notable and novel aspect of this study was the evaluation of ADSC-derived EVs. Our results demonstrated that these vesicles significantly downregulated CYP1A1 expression and AhR activity, as indicated by both reduced GFP fluorescence and lower mRNA levels. EVs are increasingly recognized as mediators of intercellular communication, and studies have shown they can carry CYP enzymes or microRNAs that influence detoxification pathways [33,34]. In our model, the EVs likely delivered regulatory molecules that interfered with AhR signaling or gene transcription, pointing to their potential role in modulating xenobiotic metabolism in liver cancer cells. This finding expands the current understanding of how stem cell-derived EVs might contribute to hepatoprotection or tumor suppression via gene regulatory pathways.

Moreover, the literature suggests that EVs could also serve as delivery systems for bioactive compounds such as curcumin, potentially enhancing their stability and bioavailability while preserving AhR-modulating activity [34]. These synergistic or additive effects between EVs and phytochemicals like curcumin warrant further exploration, especially given the overlapping targets in the AhR signaling pathway. The CAFLUX system is well-positioned to support such future investigations, as it allows concurrent assessment of transcriptional, morphological, and dose–response dynamics in a cancer-relevant context.

This study highlights the utility of the CAFLUX HepG2 cell line as a sensitive and high-resolution platform for screening environmental toxins and bioactive agents that modulate the AhR–CYP1A1 signaling axis. By integrating real-time imaging of nuclear GFP expression with transcriptional readouts, the system enables dynamic monitoring of receptor activity and its downstream effects. Its applicability in high-content screening, mechanistic toxicology, and pharmacological evaluation makes it a valuable tool for both basic research and translational studies in liver disease and oncology. The observed correspondence between fluorescence intensity and cellular responses, such as apoptosis, further underscores its potential in assessing compound-specific toxicodynamic profiles.

Despite its strengths, several limitations should be acknowledged. First, although consistent patterns were observed between GFP signal and CYP1A1 mRNA levels in response to AhR modulators, a quantitative correlation analysis across a range of concentrations was not performed, leaving the precise relationship between reporter activity and gene expression undefined. Additionally, protein-level validation (e.g., CYP1A1 Western blotting) was not included, which would have strengthened mechanistic interpretations. While the monoclonal CAFLUX line was generated via lentiviral transduction and single-cell cloning, potential variability in transgene expression among subclones was not systematically evaluated.

Regarding the ADSC-derived EVs, although they demonstrated inhibitory effects on AhR activity and CYP1A1 expression, the active molecular constituents (e.g., miRNAs, proteins, or lipids) responsible for these effects were not identified. Moreover, experiments were conducted at a single concentration, limiting insight into dose-dependency or potency thresholds. Further studies involving EV cargo profiling and functional validation, as well as dose–response analyses, are essential to elucidate the underlying mechanisms and fully realize the potential of the CAFLUX system in EV-based toxicological and therapeutic investigations.

## 4. Materials and Methods

### 4.1. Gene Preparation and Plasmid Digestion of pFUGW

The XRE-containing promoter and H2B gene were cloned from the donor plasmid DREs-H2B-eGFP (Addgene #182294) using a primer pair containing the *Pac*I and *Bam*HI enzymes (BioLabs England). This fragment 1646 bp includes a promoter region harboring DRE motifs and a protein histon (H2B) coding sequence. The fragment was flanked by *Pac*I and *Bam*HI restriction sites at its 5′ and 3′ ends, respectively. The pFUGW plasmid (Addgene #14883, Watertown, MA, USA) was linearized using the restriction enzymes *Pac*I and *BamH*I to generate a cloning vector pFUGW for insertion of the DREs–H2B-EGFP gene construct. The digestion reaction mixture included 15 µL of pFUGW plasmid, 3 µL *Pac*I, 3 µL *Bam*HI, 5 µL reaction buffer, and 24 µL nuclease-free water, incubated overnight at 37 °C. Following digestion, both the DREs–H2B insert and the linearized pFUGW (included EGFP) vector were purified using a DNA gel extraction kit (Thermo Fisher Scientific, USA) for downstream ligation.

### 4.2. Lentiviral Vector Construction

The DREs-H2B gene fragment was ligated into the linearized pFUGW (included EGFP) vector using T4 DNA ligase. The 10 µL ligation reaction included 3 µL of gene insert, 3 µL of digested pFUGW vector, 1 µL of ligase buffer, 1 µL of T4 DNA ligase (BioLabs, Watertown, MA, USA), and 2 µL of nuclease-free water, incubated for 1 h at 22 °C. The ligation product was transformed into E. coli DH5α competent cells using the heat-shock method. Transformed cells were plated on LB agar containing 100 µg/mL ampicillin and incubated overnight at 37 °C. Plasmids were isolated from bacterial colonies using the mini-prep method from a prior study [35]. Insert confirmation was performed by double digestion with *Pac*I and *Bam*HI followed by Sanger sequencing [36].

### 4.3. Large-Scale Plasmid Preparation

For lentivirus production, large-scale plasmid extraction was performed. Recombinant E. coli DH5α harboring the pFUGW/DREs-H2B-EGFP plasmid was cultured overnight at 37 °C, 200 rpm in 100 mL LB broth supplemented with 100 µg/mL ampicillin. Cells were harvested, and plasmid DNA was extracted using the QIAgen Plasmid Midi Kit. Plasmid concentration and purity were assessed using 1% agarose gel electrophoresis and quantified by NanoDrop spectrophotometry (Thermo Fisher Scientific, USA).

### 4.4. Lentivirus Packaging and Gene Transfer Using Lipofectamine 3000

Lentiviral particles containing the DREs-H2B–EGFP construct were produced using HEK 293FT cells (Invitrogen, Carlsbad, CA, USA) following a modified protocol from the MD Anderson Cancer Center. HEK 293FT cells (3.5 × 10^6^) were seeded into 6-well plates (10 cm^2^ surface area) in DMEM medium without antibiotics. After reaching ~90% confluency, cells were co-transfected with three plasmids: FUGW–DRE–EGFP, pCMV-dR8.2 dvpr, and pCMV-VSV-G in a 5:5:1 ratio using Lipofectamine 3000 (Thermo Fisher Scientific, USA) in Opti-MEM (final volume 0.5 mL per well) [37].

Transfection was carried out at 37 °C, 5% CO_2_ for six hours. The transfection medium was then replaced with 3 mL DMEM supplemented with 10% fetal bovine serum (FBS) and 1% penicillin/streptomycin (P/S). Plates were incubated for an additional 72 h at 37 °C, 5% CO_2_. Viral supernatant was collected, filtered through a 0.45 μm membrane, and concentrated using a Microsep™ Advance centrifugal filter. Virions were assessed using PCR and real-time quantitative PCR (qPCR).

### 4.5. Lentiviral Transduction of HepG2 Cells to Establish the CAFLUX Cell Line

HepG2 cells were seeded in 6-well plates at a density of 2 × 10^5^ cells per well and cultured overnight in DMEM supplemented with 10% fetal bovine serum (FBS). Upon reaching 60–70% confluency, cells were transduced with lentiviral particles encoding the DREs-H2B–EGFP construct at a multiplicity of infection (MOI) of ~10, in the presence of 8 µg/mL polybrene to enhance transduction efficiency. After 24 h, the medium was refreshed, and cells were incubated for an additional 48–72 h.

Transduced cells were expanded through 1–2 passages and screened for nuclear GFP expression. Stable monoclonal lines were established by limiting dilution and selected based on the intensity and uniformity of GFP fluorescence. The clone exhibiting consistent nuclear expression and strong responsiveness to TCDD was designated as the CAFLUX HepG2 cell line for subsequent studies.

### 4.6. Assessment of Transgenic Cell Lines Using Standard Compounds

To evaluate the responsiveness of the reporter system to standard dioxin-like compounds, transfected cells were seeded into 96-well plates at a density of 1 × 10^4^ cells per well in 100 µL of DMEM supplemented with 10% FBS and 1% penicillin/streptomycin, and incubated at 37 °C in 5% CO_2_. After 24 h, cells were exposed to serial dilutions of 2,3,7,8-tetrachlorodibenzo-p-dioxin (TCDD, 0.0001–1 pM) or benzo[a]pyrene (B[a]P, 0.00001–10 pM). Control wells received equivalent volumes of DMSO matching those of the experimental groups. All treatments, including controls, were performed in triplicate. After 24 h of exposure, GFP fluorescence intensity was measured using a Synergy HT Microplate Reader (BioTek, Winooski, VT, USA) at excitation/emission wavelengths of 485/515 nm.

### 4.7. Determination of Detection Limit

The detection limit was defined as the lowest concentration of analyte that yielded a positive result in at least 90% of replicates. Serial dilutions of TCDD and B[a]P were prepared and applied to the transgenic cells. At the lowest concentration where a positive fluorescent signal was still observed, the experiment was repeated 10 times. A detection threshold was confirmed if ≥90% of replicates yielded a positive response, indicating the assay’s sensitivity meets or exceeds the defined threshold for the reference standard.

### 4.8. Isolation and Characterization of ADSC-Derived Evs

Human adipose-derived mesenchymal stem cells (ADSCs; Lonza, PT-5006) were cultured in serum-free medium for 48 h. The conditioned medium was collected and filtered through a 0.22 µm membrane to remove debris, followed by EV isolation via ultracentrifugation. The EV pellet was washed and resuspended in sterile PBS. Nanoparticle Tracking Analysis (NTA) was performed using a ZetaView^®^ PMX-230-S-488/660 system (Particle Metrix, Germany) to assess particle size and concentration. Samples were diluted in PBS, filtered again, and analyzed at room temperature under standard settings. Fluorescent NTA was also conducted to visualize labeled EVs.

### 4.9. Assessment of Curcumin and ADSC-EVs on CAFLUX HepG2 Cells

To evaluate AhR pathway modulation, CAFLUX HepG2 cells were seeded at 1 × 10^4^ cells/well in black 96-well plates and cultured for 24 h. Cells were treated with curcumin (0–50 μg/mL) dissolved in DMSO (final DMSO ≤ 0.1%) or with ADSC-derived EVs at serial dilutions (0–100% *v*/*v* in serum-free medium). After 24 h, nuclear GFP fluorescence was measured using a Biotek Synergy HT reader. GFP expression was normalized to vehicle controls, and fluorescence images were captured to visualize nuclear signals.

### 4.10. CYP1A1 Gene Expression Analysis by qPCR

To confirm transcriptional changes, CAFLUX HepG2 cells were treated with benzo[a]pyrene (B[a]P, 1 pM), TCDD (1 pM), curcumin (30 μg/mL), or ADSC-derived EVs (40%). Total RNA was extracted using Easy-BLUE™ (iNtRON, Korea), and 1 μg of RNA was reverse-transcribed with the Maxime™ RT PreMix Kit. Quantitative real-time PCR was then performed using SYBR Green chemistry on a MyGo Pro system (QuantaBio, Beverly, MA, USA), with GAPDH as the internal control. Relative gene expression levels were calculated using the 2^−^ΔΔCt method.

### 4.11. Primer Sequences

- CYP1A1-F: 5′-GATTGAGCACTGTCAGGAGAAGC-3′
- CYP1A1-R: 5′-ATGAGGCTCCAGGAGATAGCAG-3′
- GAPDH-F: 5′-TTGTCTCACTTGTTCTCT-3′
- GAPDH-R: 5′-ATGGGAGTTGTTTTCTTG-3′

### 4.12. Statistical Analysis

Statistical analysis and image processing were performed using Microsoft Excel and ImageJ software (https://imagej.net/ij/download.html, accessed on 10 October 2025). Data are presented as mean values with appropriate statistical representation where applicable.

## 5. Conclusions

In summary, this study successfully established a stable CAFLUX HepG2 reporter cell line, offering a sensitive and robust platform for monitoring AhR activity via a histone–GFP fusion construct. The system effectively detects both AhR agonists and antagonists, with nuclear-localized GFP expression enabling real-time fluorescence visualization of transcriptional responses. Its high specificity, reproducibility, and imaging capability make it a valuable tool for mechanistic toxicology, compound screening, and studying the regulatory roles of EVs in liver cancer and related signaling pathways.

## Data Availability

Data are available upon reasonable request. Contact correspondence.
